# Real-state of autophagy signaling pathway in neurodegenerative disease; focus on multiple sclerosis

**DOI:** 10.1186/s12950-020-0237-8

**Published:** 2020-02-11

**Authors:** Mehdi Hassanpour, Fateme Hajihassani, Amirataollah Hiradfar, Nasser Aghamohammadzadeh, Reza Rahbarghazi, Nasser Safaie, Mohammad Nouri, Yunes Panahi

**Affiliations:** 1 Department of Clinical Biochemistry and Laboratory Medicine, Tabriz University of Medical Sciences, 5166614756, Imam Reza St., Golgasht St, Tabriz, Iran; 2 Stem Cell And Regenerative Medicine Institute, Tabriz University of Medical Sciences, Tabriz, Iran; 3 Clinical Pharmacy Department, Faculty of Pharmacy, Baqiyatallah University of Medical Sciences, Tehran, 1435916471 Iran; 4 Department of Health Management, School of Management and Medical informatics, Tabriz University of Medical Sciences, Tabriz, Iran; 5 Pediatric Health Research Center, Tabriz University of Medical Sciences, Tabriz, Iran; 6 Endocrine Research Center, Tabriz University of Medical Science, Tabriz, Iran; 7 Stem Cell Research Center, Tabriz University of Medical Sciences, Tabriz, Iran; 8 Drug Applied Research Center, Tabriz University of Medical Sciences, Tabriz, Iran; 9 Cardiovascular Research Center, Tabriz University of Medical Sciences, Tabriz, Iran

**Keywords:** Neurodegenerative disease, Autophagy, Multiple sclerosis

## Abstract

The occurrence of neurodegenerative disease is increasingly raised. From physiopathological aspect, the emergence of auto-reactive antibodies against the nervous system antigens contributes to de-myelination in Multiple sclerosis (MS). These features cause the nervous system dysfunction. The follow-up of molecular alterations could give us a real-state vision about intracellular status during pathological circumstances. In this review, we focus on the autophagic response during MS progression and further understand the relationship between autophagy and MS and its modulatory effect on the MS evolution. The authors reviewed studies published on the autophagy status in neurodegenerative disease and on the autophagy modulation in MS prognosis, diagnosis, and possible therapies. The inevitable role of autophagy was shown in the early-stage progression of MS. Due to critical role of autophagy in different stage of cell activity in nervous system, the distinct role of autophagy should not be neglected in the development, pathogenesis, and treatment of MS.

## Introduction

### Overview of autophagy

The balance between protein anabolism and catabolism is a fundamental mechanism for the normal function of each type cell of body [1]. Cells commonly exploit two main approaches to eliminate dysfunctional proteins, including an ubiquitin-proteasome system and autophagy process [2]. From an evolutionary perspective, autophagy is conserved and regulated cellular catabolic molecular interaction that is required for cell bioactivity during differentiation, growth, proliferation, and starvation [3]. Autophagy is termed as inclusion of misfolded, impaired, and toxic aggregate-prone mutant proteins, whole dysfunctional organelles, or intracellular pathogens into double-membrane autophagic vesicles namely autophagosomes further fuse with lysosomes to form autophagolysosome that is essential for enzymatic degradation of target components [4]. Based on the route of molecules delivery to the lysosomes, three distinct kinds of autophagy mechanisms were introduced: (**a**) In the common and frequent form of autophagy response termed macroautophagy, the cargo were enclosed with a dual lipid membrane autophagosomes to fuse with lysosomes (**b**) In the form of micro-autophagy, target components directly enters components into the lysosomes via the engulfing of self-membrane (**c**) and the last form named chaperon-mediated autophagy (CMA) that uses specific motifs (KFERQ: Lys-Phe-Glu-Arg-Gln) for the degradation of a proteins with collaboration of HSC70 complex and then adhere to lysosomes via lysosome-associated membrane protein 2A (LAMP2A). Moreover, the autophagic signaling is also triggered by mitochondrial-related axis (mitophagy) which is responsible for the removal of injured and aged mitochondria. Other aliases exist regarding autophagy such as axonophagy, lipophagy, and xenophagy based on which substance is sequestrated and digested [1]. In this review, macroautophagy will be termed as autophagy.

### Effectors participated in autophagy machinery

Autophagy is activated in response to multiple external and internal stimuli. Phosphorylation of the AMPK and inhibition of mTOR is at the center of autophagic activity of each cell. Almost 30 autophagy-related genes (Atgs) participate in the promotion of autophagy. Autophagic flux is mainly regulated through effector namely mTOR by Unc-51-like kinase complex (ULK) activity. As above-mentioned, autophagy signaling is coincided with the formation of vesicle in the three distinct steps initiation, elongation and maturation followed with following with fusion to lysosomes. In elementary phase of vacuole formation, an initiation complex consist of three complexes (1) ULK complex with ATG1, ATG13, ATG17, and ATG9, (2) PI3 kinase complex with ATG6 (also known as beclin-1) and (3) ATG5-ATG12-ATG16 polymerization complex, will be applied. ATG1-ATG13 complex recalls the factor ATG9, an essential effector for the early lipidation of the phagophore sheath. PI3 kinase-beclin1 complex formation is based on the interaction of relevant partners which can induce/inhibit autophagy. This complex could also recruit other essential ATG proteins required for the development of phagosomes. UV resistance-associated gene (UVRAG) in association with activating molecule in Beclin 1 regulated autophagy (AMBRA) and ATG14 enhances autophagy via beclin-1 complex interaction. Contrary to this, UVRAG-RUBICON complex leads to autophagy suppression. Soon after autophagy induction, beclin-1 is released from Bcl2 (B-cell lymphoma 2) located at the endoplasmic reticulum (ER), then forms complex with UVRAG/AMBRA to trigger ATG5-ATG12-ATG16 polymeric complex formation by applying factors ATG7 and ATG10. Afterwards, vacuolar membrane is enriched with Microtubule-associated proteins 1A/1B light chain 3B (MAP 1LC3), hereafter referred to as LC3, originated from cleavage and lipidation of LC3-I (ATG8) by ATG4.

**Fig. 1:**
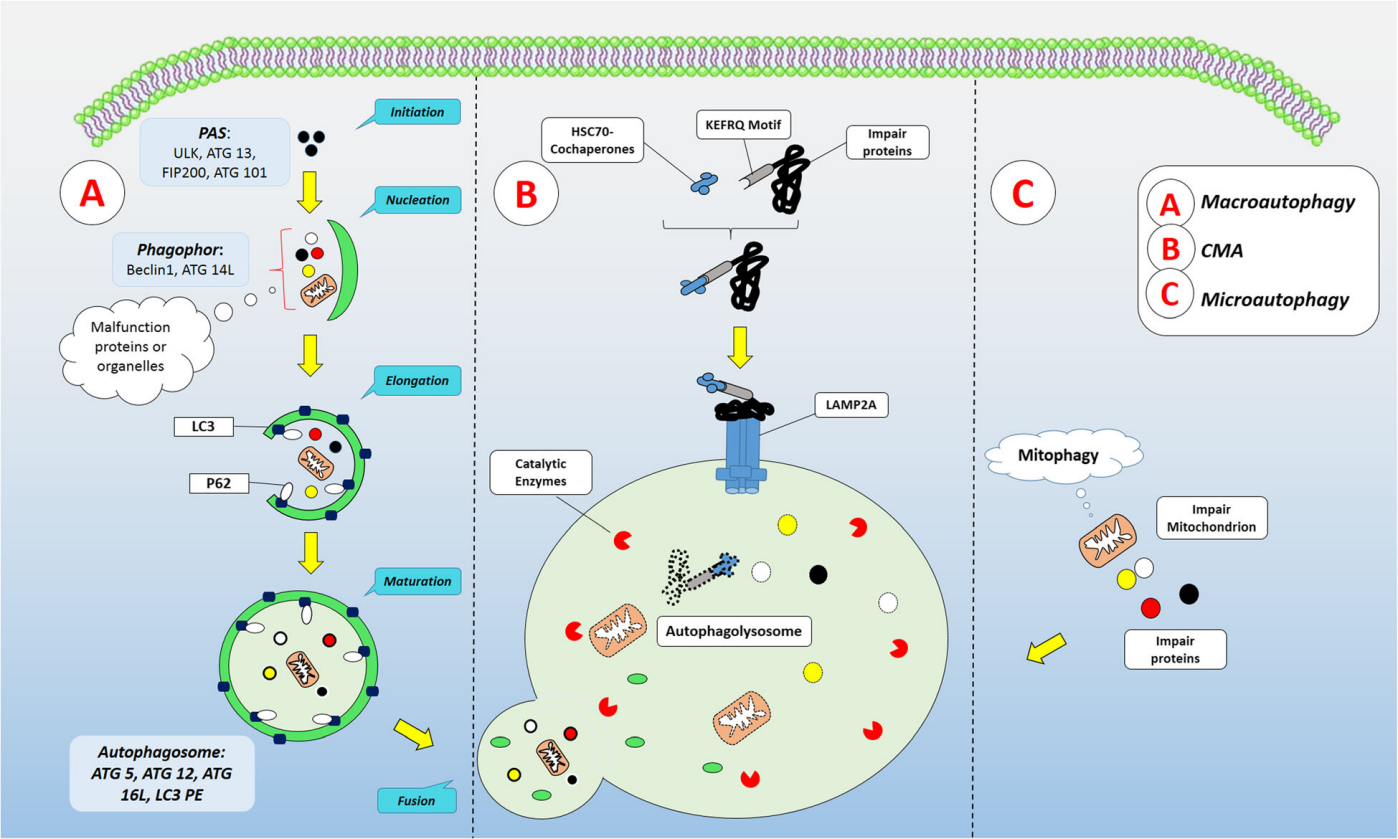
An overview of autophagy machinery. Autophagy, based on route of delivery, three different kinds of autophagy mechanisms were happen

The target molecule is conjugated with phosphatidylinositol (PE) and distributed on both sides of the membrane by ATG9 activity [5]. During elongation and formation of autophagosomes, the recruitment of targeted molecules is well documented. The completion of autophagosomes formation is fulfilled by the release of LC3bII from the external surface of the double-membrane and further recycled. Therefore, the dynamics of LC3bII molecule is prominent biomarker to monitor autophagy status. The newly formed autophagosome together with the selected cargo for degradation fuses with lysosomes to form autophagolysosome (also named autolysosome or amphisome). Cytoskeletal microtubules transfer push autophagosomes to lysosomal proximity by the help of lysosomal membrane proteins LAMP1/2 and Rab7, member of Rab family GTPases and vesicular proteins such as class III Vps (vacuolar morphogenesis proteins), SNARE (soluble NSF attachment protein receptors) and ESCRT (Endosomal Sorting Complex Required For Transport). The stages of autophagy pathway have been summarized in Fig. 1.

### Axonophagy

This kind of autophagy is commonly occurred in nervous system. Axonophagy or neuronal autophagy is defined as selective axonal degradation under pathological conditions in central nervous system (CNS) and spinal cord neurodegenerative disease including Parkinson, Alzheimer, Huntington, amyotrophic lateral sclerosis and multiple sclerosis (MS). The axonal degradation is initiated during early period of degenerative disease. To highlight the role of autophagy response, Yue et al. declared that axonal dystrophic terminals in depredating neurons contained a large number of autophagosomes [6]. According to neuron morphology and structure, neuronal autophagy is commonly seen and/or constitute in axons more than in the cell soma or dendrites after exposure to toxic insults. The formation of autophagosomes in distal axons is promoted by the regulated assembly of Atg proteins and then recruited to the endoplasmic reticulum in soma [7]. Although it has been elucidated that the majority of autophagosomes in distal axons are somewhat in a same maturation state compared to the soma-derived autophagosomes and they are at the different stages of maturation [8]. In support of this claim, cell imaging of fluorescent-labeled autophagosomes inside cerebellar granule neurons revealed a retrograde transport from axonal ends to soma while carrying different cargoes. The process of autophagosomes is preceded via three distinct pathways through microtubules, the dynein/dynactin complex and Kinesin-1. As the intensity of insults increases, the induction of autophagosomes formation exceeds the cell clearance activity, thus are accumulated inside the neurons. The occurrence of axonal swelling causes cytoskeletal disturbance and the interruption of intracellular vesicular transport. These changes contribute to the accumulation of autophagosomes in axonal ends.

It is mentioned that the promotion of autophagy response could reverse the progression pathological changes in axons. However, the autophagy signaling pathway effectors such as Beclin-1 are inactivated by induced myeloid leukemia cell differentiation protein-1 (Mcl-1) under normal conditions. After the initiation of axonal degeneration, termed Wallerian degeneration, the phosphorylation of glycogen synthase kinase 3B (GSK3B) decreases Mcl-1-mediated inhibition of Beclin-1.The activation of autophagy seems critical to afford energy demand for the completion of Wallerian degeneration. Notably, the inhibition of Mcl-1 and GSK3B causes the abortion of autophagy response and cellular degeneration against Wallerian degeneration. Other autophagy factors such as p62/SQSTM1 is elevated after the impairment of autophagic degradation [9]. Therefore, monitoring the level of autophagic signaling pathways is useful to forecast progression and/or inhibition of autophagic response. In addition to engagement of autophagic element to precede the vesicular transport, calcium influx also participate during the occurrence of axonopathy [10]. Following the completion of autophagic response, autophagosomes are released to the extracellular microenvironment. Knöferle et al. claimed that the formation of autophagosomes followed by axonophagy process which is dependent on calcium ion content and translocation. In some cases, the calcium level to promote these changes is adsorbed from out of the cells [11]. Mechanical damage to the optic nerve induced entry of extracellular calcium into axolemma via calcium channels, resulting in rapid escalation of Ca^2+^ levels. Ca^2+^ incrassation lead to secondary generation of autophagosomes and axonal degeneration.

### Relationship between autophagy state and neurodegenerative abnormalities

**Fig. 2:**
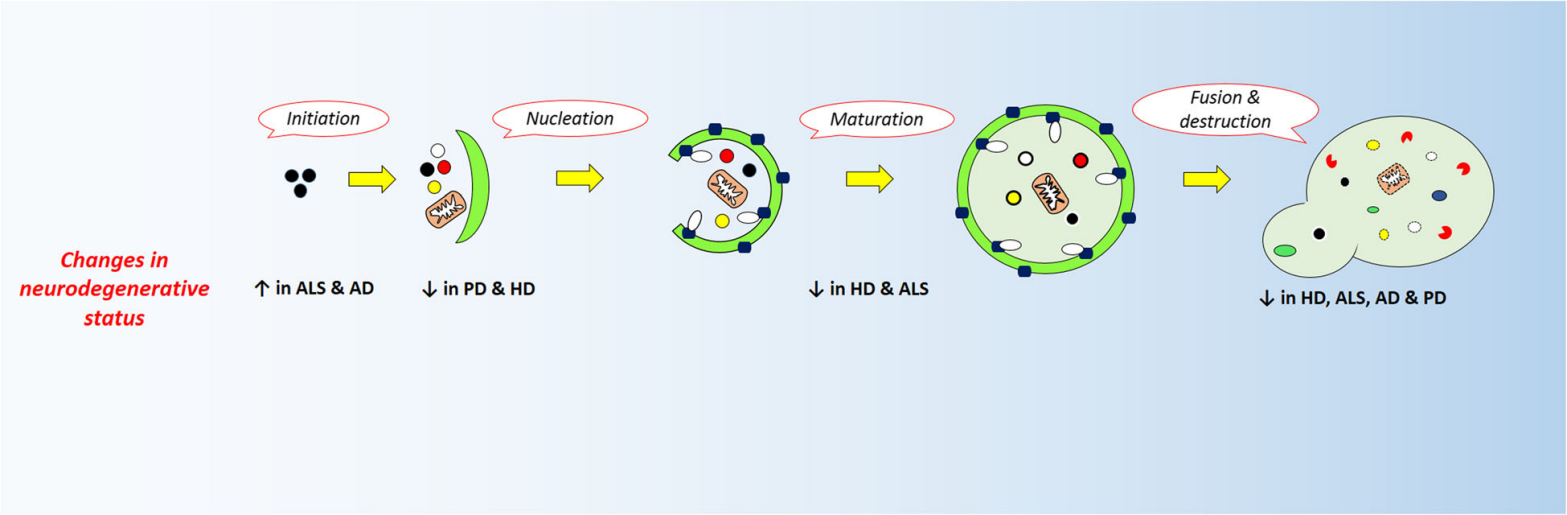
Effect of impaired phases of autophagy in different neurodegenerative contexts. This graphic diagram demonstrates the all dysfunctional steps of autophagy, from initiation to cell membrane fusion, and its relation to neurodegenerative diseases. Symbols ↑ or ↓ stand for the induction or inhibition of autophagy, respectively. ALS: Amyotrophic lateral sclerosis; PD: Parkinson disease; HD: Huntington disease; AD: Alzheimer’s disease

As aforementioned, the close relation is linked between autophagy and various neurodegenerative diseases [12–15]. The suppression of autophagy via different effectors such as mTOR promotion increases pro-inflammatory response of microglia that contributes to progression of nervous system degeneration. In contrast, the use of rapamycin for the inhibition of mTOR factor and autophagy activation has beneficial therapeutic effects in patients with MS [16]. Therefore, a possible explanation for these results is that mTOR blockers could reduce neuro-inflammation by the suppression of microglial activity and decrease of pro-inflammatory cytokines [17]. Interestingly, the malfunction of autophagy signaling results in a large number of neurodegenerative abnormalities. The aggregation of specific proteins ensues conformational disorder and is one of the hallmarks during the promotion of neurodegenerative changes, causing dementia and movement disorder. Then, degradative autophagy process removal could lead to neuronal cell death and mortality [12]. Activated autophagy potentially omits neurodegenerative associated proteins such as mutant amyloid-β peptides, hyper phosphorylated tau proteins, amyloid precursor protein (APP), Lewy bodies components and α-synuclein, huntingtin, type 1 superoxide dismutase, Alsin Rho Guanine Nucleotide Exchange Factor 2, and optineurin [18–21]. The severity of the disease closely correlates with the content of impaired proteins [22]. In this regard, genetically dysfunctional autophagic flux in the CNS caused in such proteinopathies, suggests a direct link between the autophagy activity and neurodegenerative conditions [23–25]. Different neurodegenerative disease manipulate different steps of autophagic flux illustrated in Fig. 2**.**

Here, we discuss the evidence suggesting that autophagy malfunction may contribute to the development and progression of MS, the underlying mechanisms that link autophagy with various aspects of MS pathology.

### Critical role of autophagy in neural function and dynamic

Autophagy is activated in the physiologic activity of neurons and even under the pathophysiologic conditions such as neuropathies and genetic disorders. Following the activation of autophagy, malfunction molecules and organelles are excluded. In contrast, uncontrolled autophagy activity could lead to neurodegeneration. The hypothesis that autophagy may participate in cell death in neurodegenerative status, has recently appeared and is one of hot topic investigations.

Studies have been shown that numbers of autophagic/autolysosomal vacuoles have increase after axonal damage, cytotoxin exposure, in genetic models of degeneration, and neurodegenerative disease. In neurodegenerative disorders, impairment at distinct stages of autophagy results in the accumulation of damaged organelles or pathogenic proteins. Meanwhile, autophagy is widely considered as both a vital homeostatic mechanism in healthy neuronal cells and a cyto-protective compensatory response during chronic neurodegenerative abnormalities. In this regard, Koike et al. documented evidence of complete neuronal cell death needing autophagy in in in vivo condition. Autophagic cell death is characterized by apoptosis/necrosis-independent death mechanisms, an increased autophagic flux in the dying neurons, and the prevention of cell death by autophagy suppression [26]. In some cases, a neuronal cell death involving autophagy reported in lysosomal storage disorders. In neurodegenerative disorders, deficiencies at distinct steps of autophagy can trigger neuronal cell death in several ways. In pathological conditions, abortion of autophagic response led to an increased mitochondrial injury, promoting apoptosis. In conditions with reduced autophagic degradation rate of proapoptotic factors, such as Caspases, cells enter to apoptotic condition [27].

### Autophagy and MS

In many cases, MS is considered as an autoimmune disease characterized by neuro-inflammation and demyelination, afflicting near to 2.5 million people worldwide. The clinical forms of MS include relapsing-remitting MS (RRMS), secondary progressive MS (SPMS), primary progressive MS (PPMS), and progressive relapsing MS (PRMS). The occurrence of demyelination in MS is mediated by the activity of immune T cells. It seems that the prolonged survival of auto-reactive T cells in MS is as a main cause of inflammatory cascades, leading to myelin loss and clinical relapse. Recently, it has been shown that T cell survival is modulated by the activity of Atgs. MS is a chronic multi-dimensional demyelinating neurodegenerative disease of the CNS that assumed inflammation-related and autoimmune origin. In light of clinical signs, MS patients are recognized by a spatial and temporal insufficiency originated from multifocal injuries in the periventricular white matter and by an immunoglobulin synthesis within the CNS. Proliferation and survival of T cell have been associated with the intensity of MS [28]. It is reported that expression of T cell CD46 surface marker is modulated in MS. The critical role of CD46 has been detected to participate in many biological responses including both inflammation and autophagy. Hence, it can be concluded that one of the autophagy inducing pathways in MS patients is associated with the marker CD46 [29].

### Autophagy and MS in the peripheral immune system

Autophagy participates in both innate immunity and adaptive immune system. The regulatory role of autophagy in the long-term survival of adaptive immune cells has recently surfaced as a defect in some autoimmune disease such as MS and rheumatoid arthritis [30, 31]. It was revealed that T helper1 cells (Th1) and Th17 cells have a central role at the early and later phase of MS pathogenesis. Autophagy is necessary for the reduction of auto-reactive T-cells. This pathway contributes to several functions of the adaptive immune system, including antigen presentation and T-cells homeostasis. As well-known, the cells involved in the launch and progression of inflammation processes are CD4 positive T cells, CD8 positive T cells, γ/δ T cells, B cells, and cells of the innate immune system such as microglia and macrophages [32]. Autophagy develops antigen presentation to CD4 positive T-cells via MHC II molecules [33]. In patients that suffer from MS, ATG5 protein levels are upregulated in infiltrating T cells to inflammatory sites. Additionally, it has been shown that knockdown of thymic specific Atg5 in mice experimental model lead to infiltration of auto-reactive CD4^+^ T-cells into multiple organs [34]. Moreover, blockage of autophagy in dendritic cells by Chloroquine diminishes the severity of experimental autoimmune encephalomyelitis [35]. Concurrently, Cooney et al. demonstrated that the ablation of Atg16L1 in dendritic cells during Crohn’s disease resulted in defective antigen presentation [36]. Furthermore, it has been stated that single nucleotide polymorphisms in key genes of autophagy such as Atg7 and Atg16L1, can be potentially responsible for the subsequent impact on adaptive immune cells prolonged survival [37]. While long-term survival of auto-reactive adaptive immune cells is a straightforward disease-promoting abnormality. Maria Liguori and coworkers found these two genes significantly dysregulated in MS compared to the control group [38]. Autophagy modulates the biological process such as differentiation, maturation, proliferation, cell death and homeostasis of T cells during adaptive immunity [39, 40]. Additionally, prolonged T-cell survival and increased T-cell proliferation capacity have been linked to disease relapse and progression in MS. In experimental autoimmune encephalitis (animal model mimicking MS), autophagy catabolizes pro-caspase enzymes in T cells and antagonizes apoptosis that implicate autophagy in the survival rate of auto-reactive T cells. Moreover, studies have demonstrated that ATG5, as a deputy of autophagy, participates in T cell survival regulating the differentiation, maturation and proliferation of CD4/8 positive T- and B-cells [40, 41]. In validation of this issue, Delgoffe et al. demonstrated that mTOR-ablation in T cells inhibit differentiation into Th1, Th2, and Th17 cells [42]. It was shown that the level of Atg5 is impressively increased in T-cell from MS mouse models compared to control subjects. The elevated autophagy in T-cells may enhance their survival and contribute to the MS pathogenesis [28]. Yin and colleagues revealed that Atg16L2 may have an important role in the autophagic response of T lymphocytes and serve as a promising biomarker to predict clinical relapse of MS. They showed a reduced Atg16L2 mRNA expression in T cells of MS subjects, reflecting the irregular activation of T cells. The reduction of Atg16L2 may result in malfunction of the Atg12-Atg5-Atg16 complex and preventing its localization to the pre-autophagosomal structure (PAS), thus interrupting autophagy in T cells. They also hypothesized a probable relationship between Atg16 and active MS disease [43]. Genetic susceptibility regarding autophagy has been proposed for MS. This abnormality results from inflammation-induced demyelination of CNS. Therefore, T cells are the main pathogenic effectors in this condition [44]. Indirect causes could impair autophagic activity and favor chronic inflammation in these contexts. Alirezaei et al. has disclosed in a human study, that Atg5 overexpression in serum and peripheral T cells is associated with the vigorous RRMS symptoms, proposing that Atg5 may be used as a possible therapeutic target for MS treatment [28]. A greater understanding of Atg5 function in T cells will help us in determining the underdoing mechanisms of MS. For clarification of the function of Atg5, investigations have demonstrated that neuron specific knockout of ATG genes results in accumulation of dysfunction proteins in autophagy deficient neurons, in turn, contributing to neurodegeneration status [45, 46] .This data suggests that autophagy plays a key role in the alteration of host innate immune responses and pro-inflammatory response in CNS.

### Protective role of autophagy in MS

Malfunctioned mitochondria generate reactive oxygen species (ROS) that participate in demyelination process and axonal damage [47]. Mitophagy clears depolarized mitochondria and hamper the ROS excessive production which is protective in MS [48]. It is assumed that autophagy may be a therapeutic target for neurodegenerative diseases treatment because of its protective role [49]. For example, the up-regulation of autophagy by mTOR inhibitors such as rapamycin protects the cells against neuro-degeneration in mice model [50]. Feng et al. disclosed that defective autophagy is linked to neuronal injury in a mouse model of MS [51]. The protective effect is not only a function of autophagy releasing fuels for cells, but also appears to be associated to decrease in the amount of intracellular mitochondria (mitophagy rate). In turn, this leads to less liberate of toxic molecules such as cytochrome C and ROS from mitochondria in response to proapoptotic signals. Depletion of critical factors for autophagy induction like Atg5, Atg7 or FIP200 induces neuronal cell death and cytoplasmic accumulation of organelles or ubiquitinated proteins [52].

### Pathological role of autophagy in MS

Autophagy of nervous system is related to severe neurodegenerative conditions. As above mentioned deficient autophagy in neuronal cells results in protein accumulation and consequently the occurrence of neurodegenerative diseases. Neuro-inflammation is one of the most important actors in neurological diseases, particularly MS. Autophagy and neuro-inflammation are cross linked with each other that influences the progression of various abnormality of CNS [45]. Neuro-inflammation is inversely modulated by autophagic flux to decrease detrimental effects to the CNS. Microglia cells, main effectors of neuro-inflammation process, produce pro-inflammatory cytokines and neurotoxic elements such as ROS and NO. Under these condition, astrocytes are activated and intensify the inflammatory response which are cytotoxic on primary neurons [53]. Nevertheless, autophagy has a crucial role in maintaining neuro-inflammation at a safe level. Recently, it has been shown that the autophagy level increases in PBMC from MS during acute phase and decreases following treatment. However, the autophagy response significantly increase in RRMS and enhanced autophagy rate may play its role in the pathogenesis of MS. Patergnani et al. documented that Atg5, an autophagic marker, and Parkin, a mitophagic marker, is increased drastically in cerebrospinal fluid (CSF) of MS patients compared with healthy patients, proposing that elevated autophagy/mitophagy seems to be specifically correlated to the disease [53]. The function of autophagic cascades in MS pathophysiology is not well elucidated, and remains hesitant whether they are defensive or destructive processes. Moreover, they have been demonstrated that level of TNF-α, an inflammation marker, escalated in MS individuals, suggesting that autophagic activity and inflammation may be correlate each other. It is concluded that, with continue of investigations, serum levels of these autophagic/mitophagic molecules could be used as a diagnostic or progression biomarkers of MS [54]. Meanwhile, in other study, Igci et al. probed autophagy-related genetic profile of MS individuals and observed that many autophagy-related genes significantly over expressed in MS patients compared to healthy controls, including ULK1, ULK2, FOXO1, Bcl-2, Htt, UVRAG, Atg2B, Atg4C, Atg5, TMEM74, DAPK1, EIF2AK3, Atg11, p62, PIK3R1. However, Atg16L, Rab24, Atg9A, Fas, HGS (also known as Hrs), acid α-glucosidase (GAA), PIK3C3, AMBRA1, LAMP2, Beclin1, PIK3R4, BCL3L1, DRAM1, PIK3CA [55]. To interpret their data, the finding of Zhou and colleagues improved our understanding on why autophagy is triggered at the same time while it seems prevented transcriptionally [56]. These findings propose that some of the autophagy-related genes might have different cellular functions independent of autophagy. Nevertheless, additional investigations are required. Protein aggregates have been detected in the brain and cerebral spinal fluid of MS patients, probably due to the reduced autophagy level [57]. For a first time, Albert et al. provided ultrastructural data proposing autophagy as an underlying mechanism for synaptic pathology in chronic MS [58].

### Roles of autophagy in MS-associated demyelination and re-myelination

Autophagy is strictly related to de- and re-myelination. Rangaraju et al. demonstrated that autophagy plays powerful functions in improving Schwann cell re-myelination in mice model of demyelinating peripheral neuropathies [59]. During demyelination, microglia, as a first line defense of CNS, phagocytized cell debris that requires autophagy-related genes [60]. Loss of autophagic potency in microglia hampers the clearance of unwanted debris, resulting to impaired re-myelination and intensifies of neuro-inflammation. It has been established that autophagy inducer such as rapamycin enhanced myelination process and caused in improved survival rate of neurons in tuberous sclerosis [61]. Besides, in Long–Evans shaker rat experimental model, an exuberated autophagy flux elevated the axons myelination and myelin cover thickness during dysmyelination, indicating that the autophagy is a direct target for therapy of demyelination [62]. Meanwhile, high mobility group box chromosomal protein 1 (HMGB1), an autophagy promoter, is increased in MS patients [63]. A number of studies documented that the mTOR signaling pathway establishes the regrowth of axons in the CNS, which is important for remyelination process in MS [64]. In fact, the mechanism of autophagy in different stages of MS pathogenesis needs supplementary investigation to determine the underlying relationship between autophagy and MS Additional file 1.

## Conclusion

As above-mentioned, the modulation of autophagy could act as two-edged sword during CNS pathological conditions. Although, autophagic stimulation accelerates the release of abnormal intracellular accumulation and decrease intensity of pathologies but uncontrolled autophagic response could also force the cells to apoptotic and necrotic changes. However, the intensity and duration of autophagy modulation must be carefully monitored to precisely control cell function and bioactivity [65]. Identification of MS-specific biomarkers and relationship with autophagy response must be defined. The close association of autophagy signaling pathway with effectors playing key role in the promotion of degenerative changes needs to be clarified.

## Supplementary information

**Additional file 1.** Supplementary data for reviewers comments

## Data Availability

Not applicable.

## Abbreviations

AMBRA: Activating molecule in Beclin 1 regulated autophagy
APP: Amyloid precursor protein
Atgs: Autophagy-related genes
CMA: Chaperon-mediated autophagy
CNS: Central nervous system
CSF: Cerebrospinal fluid
ER: Endoplasmic reticulum
ESCRT: Endosomal Sorting Complex Required For Transport
GAA: Acid α-glucosidase
GSK3B: Glycogen synthase kinase 3B
HMGB1: High mobility group box chromosomal protein 1
LAMP2A: Lysosome-associated membrane protein 2A
MAP 1 LC3: Microtubule-associated proteins 1A/1B light chain 3B
Mcl-1: Myeloid leukemia cell differentiation protein-1
MS: Multiple sclerosis
PAS: Pre-autophagosomal structure
PE: Phosphatidylinositol
PPMS: Primary progressive MS
PRMS: Progressive relapsing MS
ROS: Reactive oxygen species
RRMS: Relapsing-remitting MS
SNARE: Soluble NSF attachment protein receptors
SPMS: Secondary progressive MS
Th1: T helper1 cells
ULK: Unc-51-like kinase complex
UVRAG: UV resistance-associated gene
Vps: Vacuolar morphogenesis proteins

## References

[1] Hassanpour M (2018). Distinct role of autophagy on angiogenesis: highlights on the effect of autophagy in endothelial lineage and progenitor cells. Stem Cell Res Ther.

[2] Zhao J (2015). mTOR inhibition activates overall protein degradation by the ubiquitin proteasome system as well as by autophagy. Proc Natl Acad Sci.

[3] Turksen K. Autophagy in Differentiation and Tissue Maintenance: Springer; 2019.

[4] Ghavami S (2014). Autophagy and apoptosis dysfunction in neurodegenerative disorders. Prog Neurobiol.

[5] Ghavami S (2012). Apoptosis, autophagy and ER stress in mevalonate cascade inhibition-induced cell death of human atrial fibroblasts. Cell Death Dis.

[6] Yue Z (2009). The cellular pathways of neuronal autophagy and their implication in neurodegenerative diseases. Biochimica et Biophysica Acta (BBA)-molecular. Cell Res.

[7] Maday S (2016). Mechanisms of neuronal homeostasis: Autophagy in the axon. Brain Res.

[8] Maday S, Holzbaur EL (2016). Compartment-specific regulation of autophagy in primary neurons. J Neurosci.

[9] Son JH (2012). Neuronal autophagy and neurodegenerative diseases. Exp Mol Med.

[10] Ziv NE, Spira ME (1995). Axotomy induces a transient and localized elevation of the free intracellular calcium concentration to the millimolar range. J Neurophysiol.

[11] Knöferle J (2010). Mechanisms of acute axonal degeneration in the optic nerve in vivo. Proc Natl Acad Sci.

[12] Wong E, Cuervo AM (2010). Autophagy gone awry in neurodegenerative diseases. Nat Neurosci.

[13] Frake RA (2015). Autophagy and neurodegeneration. J Clin Invest.

[14] Wong YC, Holzbaur EL (2015). Autophagosome dynamics in neurodegeneration at a glance. J Cell Sci.

[15] Kiriyama Y, Nochi H (2015). The function of autophagy in neurodegenerative diseases. Int J Mol Sci.

[16] Dello Russo C (2013). mTOR kinase, a key player in the regulation of glial functions: relevance for the therapy of multiple sclerosis. Glia.

[17] Russo CD (2009). Involvement of mTOR kinase in cytokine-dependent microglial activation and cell proliferation. Biochem Pharmacol.

[18] Rodolfo C, Campello S, Cecconi F (2018). Mitophagy in neurodegenerative diseases. Neurochem Int.

[19] Banerjee R, Beal MF, Thomas B (2010). Autophagy in neurodegenerative disorders: pathogenic roles and therapeutic implications. Trends Neurosci.

[20] Nixon RA (2013). The role of autophagy in neurodegenerative disease. Nat Med.

[21] Martinez-Vicente M (2017). Neuronal mitophagy in neurodegenerative diseases. Front Mol Neurosci.

[22] Xu J (2019). Regional protein expression in human Alzheimer’s brain correlates with disease severity. Commun Biol.

[23] Sato S (2018). Loss of autophagy in dopaminergic neurons causes Lewy pathology and motor dysfunction in aged mice. Sci Rep.

[24] López-Pérez Ó (2019). Dysregulation of autophagy in the central nervous system of sheep naturally infected with classical scrapie. Sci Rep.

[25] Liu Xiaojuan, Zhu Manhui, Ju Yuanyuan, Li Aihong, Sun Xiaolei (2019). Autophagy dysfunction in neuropathic pain. Neuropeptides.

[26] Shen Shensi, Kepp Oliver, Kroemer Guido (2012). The end of autophagic cell death?. Autophagy.

[27] Wu JJ (2009). Mitochondrial dysfunction and oxidative stress mediate the physiological impairment induced by the disruption of autophagy. Aging.

[28] Alirezaei M (2009). Elevated ATG5 expression in autoimmune demyelination and multiple sclerosis. Autophagy.

[29] Choileain SN, Astier AL (2011). CD46 plasticity and its inflammatory bias in multiple sclerosis. Arch Immunol Ther Exp.

[30] Yang Z, Goronzy JJ, Weyand CM (2015). Autophagy in autoimmune disease. J Mol Med.

[31] Consortium WTCC (2007). Genome-wide association study of 14,000 cases of seven common diseases and 3,000 shared controls. Nature.

[32] Friese MA, Fugger L (2005). Autoreactive CD8+ T cells in multiple sclerosis: a new target for therapy?. Brain.

[33] Paludan C (2005). Endogenous MHC class II processing of a viral nuclear antigen after autophagy. Science.

[34] Nedjic J (2008). Autophagy in thymic epithelium shapes the T-cell repertoire and is essential for tolerance. Nature.

[35] Bhattacharya A (2014). Deficiency of autophagy in dendritic cells protects against experimental autoimmune encephalomyelitis. J Biol Chem.

[36] Cooney R (2010). NOD2 stimulation induces autophagy in dendritic cells influencing bacterial handling and antigen presentation. Nat Med.

[37] Shaw SY (2013). Selective modulation of autophagy, innate immunity, and adaptive immunity by small molecules. ACS Chem Biol.

[38] Liguori M (2017). Combined microRNA and mRNA expression analysis in pediatric multiple sclerosis: an integrated approach to uncover novel pathogenic mechanisms of the disease. Hum Mol Genet.

[39] Jia W (2011). Autophagy regulates endoplasmic reticulum homeostasis and calcium mobilization in T lymphocytes. J Immunol.

[40] Pua HH, He Y-W (2007). Maintaining T lymphocyte homeostasis: another duty of autophagy. Autophagy.

[41] Miller BC (2008). The autophagy gene ATG5 plays an essential role in B lymphocyte development. Autophagy.

[42] Delgoffe GM (2009). The mTOR kinase differentially regulates effector and regulatory T cell lineage commitment. Immunity.

[43] Yin L (2014). Autophagy-related gene16L2, a potential serum biomarker of multiple sclerosis evaluated by bead-based proteomic technology. Neurosci Lett.

[44] Mahad DH, Trapp BD, Lassmann H (2015). Pathological mechanisms in progressive multiple sclerosis. Lancet Neurol.

[45] Hara T (2006). Suppression of basal autophagy in neural cells causes neurodegenerative disease in mice. Nature.

[46] Komatsu M (2006). Loss of autophagy in the central nervous system causes neurodegeneration in mice. Nature.

[47] Van Horssen J (2011). Radical changes in multiple sclerosis pathogenesis. Biochimica et Biophysica Acta (BBA)-Mol Basis Dis.

[48] Chen Y (2008). Oxidative stress induces autophagic cell death independent of apoptosis in transformed and cancer cells. Cell Death Differ.

[49] Rubinsztein DC (2007). Potential therapeutic applications of autophagy. Nat Rev Drug Discov.

[50] Ravikumar B (2004). Inhibition of mTOR induces autophagy and reduces toxicity of polyglutamine expansions in fly and mouse models of Huntington disease. Nat Genet.

[51] Feng X (2017). Defective autophagy is associated with neuronal injury in a mouse model of multiple sclerosis. Bosnian J Basic Med Sci.

[52] Leidal AM, Levine B, Debnath J (2018). Autophagy and the cell biology of age-related disease. Nat Cell Biol.

[53] Glass CK (2010). Mechanisms underlying inflammation in neurodegeneration. Cell.

[54] Patergnani S (2018). Autophagy and mitophagy elements are increased in body fluids of multiple sclerosis-affected individuals. J Neurol Neurosurg Psychiatry.

[55] Igci M (2016). Gene expression profiles of autophagy-related genes in multiple sclerosis. Gene.

[56] Zhao Y (2010). Cytosolic FoxO1 is essential for the induction of autophagy and tumour suppressor activity. Nat Cell Biol.

[57] David MA, Tayebi M (2014). Detection of protein aggregates in brain and cerebrospinal fluid derived from multiple sclerosis patients. Front Neurol.

[58] Albert M (2017). Synaptic pathology in the cerebellar dentate nucleus in chronic multiple sclerosis. Brain Pathol.

[59] Rangaraju S (2010). Rapamycin activates autophagy and improves myelination in explant cultures from neuropathic mice. J Neurosci.

[60] Sanjuan MA (2007). Toll-like receptor signalling in macrophages links the autophagy pathway to phagocytosis. Nature.

[61] Meikle L (2008). Response of a neuronal model of tuberous sclerosis to mammalian target of rapamycin (mTOR) inhibitors: effects on mTORC1 and Akt signaling lead to improved survival and function. J Neurosci.

[62] Smith CM, Mayer JA, Duncan ID (2013). Autophagy promotes oligodendrocyte survival and function following dysmyelination in a long-lived myelin mutant. J Neurosci.

[63] Andersson Å (2008). Pivotal advance: HMGB1 expression in active lesions of human and experimental multiple sclerosis. J Leukoc Biol.

[64] Park KK (2008). Promoting axon regeneration in the adult CNS by modulation of the PTEN/mTOR pathway. Science.

[65] Rubinsztein DC (2005). Autophagy and its possible roles in nervous system diseases, damage and repair. Autophagy.

