# Supplementary material for: Real-state of autophagy signaling pathway in neurodegenerative disease; focus on multiple sclerosis
Source: J Inflamm (Lond). 2020 Feb 11;17:6. doi: 10.1186/s12950-020-0237-8 (PMC7014934; doi:10.1186/s12950-020-0237-8)
Supplement: Supplementary file 1 — Additional file 1. Supplementary data for reviewers comments [file 12950_2020_237_MOESM1_ESM.docx]

**Point by point response to comments:**

1. Ingredients → components
2. On the other words → Contrary to this
3. Numbers → number
4. constitute commonly in → constitute in
5. As the intensity of insults increases → As the intensity of insults increases,
6. the cell clearance activity → the cell clearance activity,
7. under the normal condition → under normal conditions
8. specific proteins ensue→ specific proteins ensues
9. the hallmarks of during→ the hallmarks during
10. even → then,
11. suggesting → suggests
12. Autophagy is activated in the development, activity of neurons and even under the neuropathies and genetic disorders → Autophagy is activated in the physiologic activity of neurons and even under the pathophysiologic conditions such as neuropathies and genetic disorders.
13. Role of autophagy in regulating → The regulatory role of autophagy in
14. have central → have a central
15. involving → involved
16. process → processes
17. cells of innate immune system → cells of the innate immune system
18. ATG5 protein level has been upregulated → ATG5 protein levels are upregulated
19. Besides → Additionally
20. Also, → Moreover,
21. it has been clear that → it has been stated that
22. MS compared to control group → MS compared to the control group
23. Autophagy modulate → Autophagy modulates
24. such as differentiation, maturation, proliferation and, cell death → such as differentiation, maturation, proliferation, cell death
25. pro-Caspase → pro-caspase
26. antagonizing → antagonizes
27. studies have been demonstrated that → studies have demonstrated that
28. participate in T cell survival and regulate → participates in T cell survival regulating
29. T cells inhibit differentiate into → T cells inhibit differentiation into
30. MS model of mouse → MS mouse models
31. role in autophagic response → role in the autophagic response
32. the Atg12-Atg5-Atg16 complex and prevents its localization to pre-autophagosomal structure (PAS) interrupting autophagy in T cells. → the Atg12-Atg5-Atg16 complex preventing its localization to the pre-autophagosomal structure (PAS), thus interrupting autophagy in T cells.
33. Alirezaei et al has been disclosed in a human study → Alirezaei et al has disclosed in a human study,
34. Understanding of Atg5 function → A greater understanding of Atg5 function
35. investigations have been demonstrated → investigations have demonstrated
36. in this turn → in turn
37. autophagy play a role in alteration of host innate immune responses → autophagy plays a key role in the alteration of host innate immune responses
